# From the bench to the bedside: Breaking down the blood–brain barrier, decoding the habenula, understanding hand choice, and the role of ketone bodies in epilepsy

**DOI:** 10.4103/2152-7806.74143

**Published:** 2010-12-22

**Authors:** Jason S. Hauptman

**Affiliations:** Intellectual and Developmental Disabilities Center and Department of Neurosurgery, Geffen School of Medicine at UCLA, Los Angeles, CA, USA

## IN THIS UPDATE

- Understanding the importance of pericytes in the maintenance of the blood–brain barrier (BBB).
- Insight into the function of the habenula.
- The role of the posterior parietal cortex (PPC) in everyday actions.

A potential mechanism for why ketone bodies work in epilepsy.

## PERICYTES FOUND TO BE CRITICAL TO THE INTEGRITY OF THE BBB[2]

### Key points

1. The BBB is composed of capillary endothelium (bound together by tight junctions), astrocytic end-feet processes, and pericytes. Until recently, the role of astrocytes and pericytes as part of the BBB has been unclear.
2. Pericytes play a crucial role in regulating the integrity of the BBB by controlling transcytosis of various molecules through capillary endothelial cells.
3. Pericytes regulate the expression levels of endothelial and astrocytic genes and proteins.
4. Pericytes are also critical in ensuring that the astrocytic end-feet develop proper orientation during development, where they attach to the endothelial cells.

It is well known that the capillary endothelial cells lining the central nervous system vessels form the main physical component of the BBB through tight junctions and highly selective transport mechanisms. A study by Armulik *et al*. showed *in vivo* that pericyte integrity is crucial for the BBB to maintain selectivity to water and both small and large molecules. In order to study the role of pericytes in BBB integrity, the authors devised two mouse models that both had disrupted platelet-derived growth factor (PDGF)/PDGF receptor *β* (PDGFR-*β*) signaling. Why study PDGF/PDGFR-*β* signaling? It turns out that this signaling cascade is necessary for pericytes to find their way to their appropriate locations during blood vessel development. Because congenital deletion of PDGFR-*β* results in embryonic lethality, the researchers had to develop models in which PDGFR-*β* was disrupted in some way but was not absent. They did this in two ways. First, they developed mice that had a dysfunctional PDGFR-*β* that did not bind a specific class of extracellular molecules, heparan sulfate proteoglycans. Second, they developed another mutant model in which the native PDGFR-*β* genes were absent and instead were replaced by human PDGFR-*β* genes that could be turned “off,” specifically in endothelial cells (conditionally deleted PDGFR-*β*).

Using these models, the authors first demonstrated that the densities of pericytes were reduced in all the mutant mice, particularly in the mice with dysfunctional PDGFRs and the mice with conditional PDGFR-*β*. Associated with reduced pericyte densities was a reduced density of blood vessels that had larger diameters than usual as well as increased brain water content in the extracellular, extravascular space. This latter observation indicated a dysfunctional BBB. Altered BBB integrity was further confirmed by demonstrating that several peripheral tracers, all having different molecular weights (some large and some small) and normally excluded from crossing the BBB, accumulated within the brain parenchyma. Furthermore, the amount of tracer accumulation was inversely proportional to the pericyte density, suggesting that those with greater dysfunction in pericyte targeting suffered from greater disruption of the BBB.

Given the abnormality in BBB integrity in the pericyte-deficient mutants, the authors then turned their attention to the structure of the capillary endothelial tight junctions as they are thought to be the principal physical barrier to diffusion of molecules into the brain parenchyma. They found that despite some slight ultrastructural changes, the endothelial tight junctions appeared to have normal function. The question remained, if the endothelial cells’ tight junctions were functioning normally, where did the BBB break down in the pericyte-deficient mutants? To answer this question, the authors looked at the location of the tracers that were able to cross the BBB by examining brain slices using microscopy. They found that the tracers were located within vesicles inside the capillary endothelial cells as well as at the basement membrane. This suggested that the altered BBB integrity resulted due to an increase in transcytosis through the capillary endothelium. The researchers decided to then test imitanib, a tysrosine kinase inhibitor that has been shown to reduce edema in stroke models, on the pericyte-deficient mice. Interestingly, imitanib treatment significantly reduced tracer accumulation in the mutant animals. From their data, it appeared that imitanib functions by inhibiting the later stages of endothelial transcytosis without affecting any of the other anomalies found in the pericyte-deficient mutants.

The final aspect of this study concentrated on exploring the effect of pericyte deficiency on the expression patterns of BBB markers. To do this, the authors performed a microarray analysis (a way of looking at levels of gene expression in cells) on fragments that contained blood vessels, pericytes, and astrocytic end-feet. Their results suggested that the deficiency in pericytes had a direct effect on the levels of BBB-specific genes and proteins. Several of these genes were specific to astrocytes. As it turns out, on closer examination, the astrocytic end-feet that help to comprise the BBB showed abnormal orientation. Furthermore, some of the end-feet were completely detached from the endothelial cells that they are normally adherent to. Thus, pericytes appear to be necessary for the correct cueing of astrocytic end-feet during development. This, coupled with the notion that pericytes play a crucial role in the regulation of transcytosis in endothelial cells, sheds important new light into our understanding of BBB structure and function. Like imitanib, future pharmacologics for a variety of pathologies (tumors, vascular lesions, etc.) will either be targeted at the BBB or require adjunctive targeting of the BBB for maximal effectiveness.

## DECIPHERING THE FUNCTION OF THE HABENULA[1]

### Key points

1. The habenula is highly conserved through evolution, allowing its function to be studied in phylogenetically lower animals such as the zebrafish.
2. Neurons of the lateral habenula project to monoaminergic neurons that are involved in responding to punishment or lack of reward thus modulating avoidance behaviors.
3. Neurons of the medial habenula project to the interpeduncular nucleus (IPN), which in turn projects to the dorsal raphe, dorsal tegmental nucleus (DTN), periaqueductal gray (PAG), and nucleus incertus. By shutting off these projections, the authors show that the medial habenula likely plays a critical role in experience-dependent behavior modification, particularly in response to fear.

The habenula, an epithalamic cell mass that is located dorsal and caudal to the thalamus adjacent to the pineal gland, is embedded in the posterior end of the stria medullaris thalami. It consists of medial and lateral subnuclei. From a surgical standpoint, it is possible that the habenula may be considered as a target for neuromodulation of neuropsychiatric diseases, including depression. Anatomically speaking, the lateral and medial habenula have different projections and potentially different roles in the modulation of behavior. It is known that the lateral habenula, through projections to neurons that produce monoamine neurotransmitters, regulates the learning of avoidance behavior (i.e., they respond robustly to unpleasant stimuli or lack of reward). The medial habenula, on the other hand, projects to the IPN of the midbrain. The IPN is located, as the name implies, between the cerebral peduncles in the midbrain. While previous studies suggest that the medial habenula is involved in the processing of fear, its precise function is still not certain.

In a study, Agetsuma and colleagues studied the function of the medial habenula structural correlate in zebrafish. Because the habenular structure has been conserved throughout evolution, it is likely that its function has been conserved as well.

First, the authors injected anterograde tracers into the IPN to examine the putative targets of IPN neurons (these tracers mark the areas that IPN neurons project to). They found that these neurons project to areas that are the mammalian correlates of the dorsal raphe, PAG, DTN, and nucleus incertus (an area adjacent to the dorsal raphe and tegmentum). Using more advanced tract-tracing techniques, the authors then found that the medial habenula equivalent could be subdivided into medial (dHbM) and lateral (dHbL) subnuclei, each with parallel reciprocal connections to the dorsal raphe equivalent and PAG/DTN/nucleus incertus equivalents, respectively. To further dissect the dHbL pathway and understand their respective roles in the modulation of fear behavior, the authors created transgenic lines of zebrafish. These zebrafish expressed differentially colored fluorescent proteins in the neurons of the dHbM and dHbL to help confirm their putative targeting. Furthermore, they used a strategy that allowed them to “shut off” synaptic transmission by the dHbL neurons by employing the tetanus toxin light chain. The tetanus toxin light chain, by interacting with proteins located at the presynaptic terminal, prevents the release of neurotransmitter into the synaptic cleft.

Using a task that taught the zebrafish to fear a particular cue (in this case a light), they found that the zebrafish with dHbL silenced exhibited significantly different fear-induced behavior than the controls. While controls reacted to the stimulus with flight behavior, the transgenic zebrafish reacted by freezing. This change in behavior seemed to be associated only with the specific response to the conditioned stimulus and not to the overall locomotive activity or tendency to explore. The freezing behavior of the “silenced” zebrafish is particularly interesting because control zebrafish also exhibited freezing – but only at the beginning when they were first introduced to the conditioned stimulus paired with the unpleasant (unconditioned) stimulus, an electric shock. After a few trials, the control zebrafish stopped freezing and began to try to evade. The dHbL-silenced zebrafish, on the other hand, continued to freeze, suggesting that the initial freezing response is not modified as the zebrafish continues to experience repeated trials. The end result of this series of experiments is the suggestion that the medial habenula, specifically the lateral subnucleus, is involved in experience-dependent behavioral modification. Deficits in habenular signaling may play a role in an individual’s ability to modify behaviors in response to stress and, thus, in neuropsychiatric diseases such as depression. In fact, deep brain stimulation of the habenula has been suggested (although based on limited evidence) as a putative target for depression.[3]

## ROLE OF THE PPC IN HAND CHOICE[5]

### Key points

1. Deciding which hand to use for routine tasks in one of the most common things a person can do. Although indirect evidence suggests that this behavior may be mediated by the PPC, definitive data is lacking.
2. When measuring reaction times during reaching tasks, it takes longer for a person to reach when they are given the option to use either hand compared with when they are told which hand to use. This supports the notion that when given freedom in hand choice, “competing” motor plans exist that may slow down the reaction time.
3. Disrupting the left PPC with transcranial magnetic stimulation (TMS) results in a significant increase in ipsilateral (left-sided) hand choice when patients are given the option to choose a hand to use for a reaching task, suggesting the left PPC may provide a competing motor plan to use the right hand that is “shut off.”

In the study by Oliveira *et al*., the authors examined a simple and routine decision that humans are constantly making – which hand to use while performing a given action. In fact, one might consider this decision so common and so simple that it is an unconscious one. A prevailing theory about hand selection involves a form of “parallel planning,” in which multiple behavior plans are processed simultaneously and compete for performance. Although the PPC has been implicated in the planning of reach behaviors and hand selection, evidence solidifying this notion has been lacking. To provide more conclusive data concerning the function of the PPC in reach planning, the authors used a reaching task in which patients were either told which hand to use or they were free to choose which hand they wanted to use. Then, TMS was used to disrupt PPC processing and they then determined the effects on the reach task.

In the first set of experiments, the authors compared reaction times for patients asked to reach for an object with a particular hand versus allowing them to choose the hand to reach with. They found that when the reaching hand was predetermined, reaction times were faster than when the patient had the ability to choose (thus signifying a “cost” in reaction time associated with free choice). This increase in reaction time, as suggested by the authors, implies the competition between competing right- and left-handed action plans. In the follow-up experiment, the authors used TMS to “block” one hemisphere’s PPC from processing hand selection. First, they found that TMS of the PPC increased reaction times in general (in a non-specific fashion). But, more interestingly, they found that stimulation of the left PPC (but not the right PPC) resulted in greater choice of the ipsilateral hand for reach tasks. This did not seem to be influenced by disruption of the parietal attention areas.

The results are interesting in that they confirm the role that the PPC plays in hand choice for reaching tasks, something that people fundamentally do every day without consciously thinking about it. As for the asymmetry of effects, the best guess is that this is a confounding finding. Because the majority of patients in the study were right hand dominant and thus would show a strong preference for using their right hand, the difference needed to show an increase in right hand selection may have been too difficult to achieve. In that way, the study may have been underpowered and biased by the lack of sufficient left hand-dominant participants. This is the most likely scientific rationale for their findings, although other possibilities exist as well. Regardless of this, the study is interesting in that it sheds light on the function of the PPC, particularly underscoring its importance in every day activity. It makes one curious as to whether patients who have had surgery in their left PPC notice that they are using their left hand more for everyday tasks.

## KETONE BODIES AND EXCITATORY NEUROTRANSMISSION[4]

### Key points

1. Fasting and the ketogenic diet are well-recognized effective forms of therapy for epilepsy. While prevailing thought is that the ketone bodies are crucial for reducing neuronal excitability, existing evidence of their function is controversial and inconclusive. This report examines a potential mechanism by which ketone bodies function to reduce seizures.
2. Vesicular glutamate transporters (VGLUTs), which are responsible for loading glutamate into presynaptic vesicles that are released into the synapse, rely on low concentrations of extravesicular chloride in order to function. Chloride is not pumped into the vesicle; instead, this chloride is thought to bind to an allosteric site (a site on the VGLUT protein that allows for regulation of its activity) on the transporter that regulates its function.
3. Acetoacetate, a prototypical ketone body, competes with chloride for the allosteric-binding site on the VGLUTs and inhibits the uptake of glutamate into the vesicles. When acetoacetate binds to this site, the result is a reduction in glutamate release into the synapse and thus less excitatory neurotransmission.
4. Acetoacetate attenuates seizure activity and increases brain glutamate in chemical models of rodent epilepsy.

In the study by Juge *et al*., the authors try to understand the mechanism behind the phenomenon that fasting and the ketogenic diet control epilepsy (and thus neuronal excitability). Ketone bodies, a by-product of fasting and the ketogenic diet, are thought to be the direct cause of the antiepileptic effect. How ketone bodies affect neural excitability, however, is still debatable. Studies have failed to show differences in systemic levels brain glutamate/GABA and the amount or activity of their receptors and transporters. There may be an effect on ATP-dependent potassium channels, but this is considered controversial. In this paper, attention is turned to the VGLUTs that are required for the release of glutamate at the synapse. In previous studies, the authors have found that VGLUT function is dependent on chloride levels (such that glutamate uptake by VGLUT into vesicles for release at the synapse is strong in the presence of low levels of chloride, but glutamate uptake is low when there is no chloride or chloride levels are too high).

First, the authors showed that the activity of the glutamate transporter VGLUT2 was directly dependent on chloride concentrations. This chloride-dependent activity appeared to be directly related to the electrochemical gradient for chloride set up across the wall of the vesicle. To see whether the chloride was actually transported by the vesicle as it took up glutamate, the authors used a strategy that allowed them to detect chloride movement by either fluorescence or radioactivity. Fascinatingly, it appeared that the VGLUT2 did not actually transport chloride even though low concentrations of chloride were necessary for glutamate uptake and neurotransmission. Then, what does chloride do? The answer is that it is an allosteric modulator. That is, chloride binds to the VGLUT2 at a site other than its active site in order to regulate its activity levels.

The fact that chloride is an allosteric modulator of VGLUT2 led the authors to their next series of experiments, which were aimed at screening for other molecules that may bind to the same allosteric site as chloride and modulate VGLUT2 activity. This screen led to the discovery of ketone bodies, such as acetoacetate (found to have the strongest effect) and 3-hydroxybutyrate, which bind to the allosteric site and inhibit glutamate transport into the vesicle. This would have the effect of reducing glutamate neurotransmission and thus reducing synaptic excitability. When testing acetoacetate in the presence of chloride, the authors found that higher levels of chloride were necessary to preserve VGLUT2 function. This suggests that acetoacetate and chloride were in effect “competing” for the same binding site on VGLUT2, with opposing effects. Perhaps the most convincing data came from their experiments looking at other types of vesicular neurotransmitter transporters. They found that the vesicular transporter for GABA was immune to acetoacetate while the transporters for the excitatory neurotransmitters (other VGLUTs, vesicular excitatory amino acid transporter) showed chloride dependence and inhibition by acetoacetate.

To verify these effects in a more physiologically relevant model, the authors stimulated neurons and astrocytes derived from the hippocampus with high chloride and found that acetoacetate inhibited glutamate release from neurons but not astrocytes. Then, using some electrophysiology methods, the authors demonstrated that acetoacetate results in a reduction in quantal spontaneous (non-action potential dependent) release of glutamate but not GABA from the hippocampal neurons in brain slices. Finally, the authors induced seizures chemically using 4-aminopyridine (4-AP) injected directly into the rat brain. While 4-AP causes seizures and increases in brain glutamate levels, the addition of acetoacetate reduced the intensity of seizures and the levels of glutamate, an effect that was reversible after removal of acetoacetate from the dialysate solution. In all, the authors provide strong evidence that ketone bodies reduce neuronal excitability by depleting the presynaptic vesicular pool of glutamate. Thus, less glutamate is released in the synapses and the postsynaptic cells become less excited. This helps to potentially explain the role of the ketogenic diet in the treatment of neuronal hyperexcitability, such as in epilepsy.
